# A critical review and update on autoimmune encephalitis: understanding the alphabet soup

**DOI:** 10.1590/0004-282X-ANP-2022-S122

**Published:** 2022-08-12

**Authors:** Mateus Mistieri Simabukuro, Guilherme Diogo da Silva, Luiz Henrique Martins Castro, Leandro Tavares Lucato

**Affiliations:** 1 Universidade de São Paulo, Faculdade de Medicina, Divisão de Neurologia, São Paulo, SP, Brazil.; 2 Universidade de São Paulo, Faculdade de Medicina, Instituto de Radiologia São Paulo, SP, Brazil.

**Keywords:** Encephalitis, Anti-N-Methyl-D-Aspartate Receptor Encephalitis, Limbic Encephalitis, Paraneoplastic Syndromes, Nervous System., Encefalite, Encefalite Anti Receptor de N-Metil-D-Aspartato, Encefalite Límbica, Síndromes Paraneoplásicas do Sistema Nervoso.

## Abstract

Autoimmune encephalitis (AE) comprises a group of diseases mediated by antibodies against neuronal cell surface or synaptic antigens, such as ion channels or neurotransmitter receptors. New clinical syndromes and their associated antibodies were and are still being characterized over the last two decades. The fact that their main clinical features are interdisciplinary, - encompassing neuropsychiatric symptoms, cognitive dysfunction, epileptic seizures, movement and sleep disorders - has led to a surge of interest in this field. Some of these diseases present with a well-defined syndrome, being recognizable on clinical grounds. Correct diagnosis is important since AE are potentially treatable diseases, despite their severity. On the other hand, an increasing number of neuronal antibodies being described casts doubt upon the way we should utilize antibody testing and interpret results. In this article we review, summarize and update the current knowledge on antibody mediated encephalitis.

## RESUMO

As encefalites autoimunes compreendem um grupo de doenças mediadas por anticorpos contra antígenos de superfície neuronal ou sinapse, como canais iônicos ou receptores de neurotransmissores. Novas síndromes clínicas e os anticorpos a elas associados foram e ainda estão sendo caracterizados ao longo das últimas duas décadas. Dado que suas principais características clínicas são interdisciplinares, isto é, incluem -se sintomas neuropisquiátricos, disfunção cognitiva, crises epilépticas, distúrbio do movimento e do sono, há uma grande onda de interesse sobre esse campo de conhecimento. Algumas dessas doenças apresentam-se com uma síndrome bem definida, sendo possível reconhecê-las clinicamente. Diagnosticá-las corretamente é importante uma vez que se trata de doenças potencialmente tratáveis apesar da gravidade que lhes é característica. Por outro lado, o número crescente de anticorpos sendo descritos causam dúvida frequente sobre qual o melhor teste a solicitar e como interpretá-los. Nós aqui apresentamos uma revisão atualização resumida do conhecimento atual sobre as encefalites mediadas por anticorpos.

## INTRODUCTION

Autoimmune encephalitis (AE) is a group of recently recognized diseases where antibodies believed to be pathogenic target neuronal proteins localized in cell surface and/or synapses, disrupting their function and provoking a peculiar symptomatology. Clinically, AE usually manifest as a combination of prominent neuropsychiatric symptoms, epileptic seizures, amnesia, movement disorders, disorders of consciousness and sleep disorders. Other than autonomic dysfunction, AE are not accompanied by systemic manifestation, such as occurs in Behçet’s disease or in systemic lupus erythematosus^1^. 

Although rare, certain AE can be easily recognized at the bedside and confirmed by antibody testing. However, these tests are not easily available and even when access is guaranteed, results can take several weeks to be obtained^2^. Expanding discovery of neuronal antibodies associated with testing technical limitations of commercial kits also poses challenges to select testing and to interpret test results. 

Recognition of autoimmune encephalitis is crucial, because AE often presents with rapidly progressive severe and debilitating symptoms which, if promptly and adequately treated, can lead to good outcomes, including full recovery in many cases. Tumor association varies depending on some factors such as antibody type, neurological syndrome and demography, influencing treatment response, relapse risk and outcomes. Future directions must address tailored treatment for each syndrome, symptomatic treatment and rehabilitation. 

This review highlights updates and controversies regarding clinical features, diagnosis and treatment of the well-known antibody-mediated encephalitis syndromes.

## EPIDEMIOLOGY

Autoimmune encephalitis is not rare: the paradigm shifts in comparison with infectious encephalitis

Data from England estimates that incidence of all types of encephalitis is 2.73 to 8.66 cases per 100,000 a year^3^. Approximately 40-50% of encephalitis cases remain without an established diagnosis^4^ ^,^ ^5^, even when patients undergo extensive testing. 

Until the discovery of neuronal cell surface antibodies, infection was believed to be the main known cause of encephalitis. However, following the characterization of anti- N-methyl-d-aspartate-receptor (NMDAR) antibodies in 2007^6^,we learned that autoimmune etiology is not rare. The California Encephalitis Project showed that in young people individuals (aged < 30), anti-NMDAR (N-methyl-d-aspartate-receptor) was the most common type of encephalitis, surpassing individual viral etiology^7^.

In the Netherlands, annual incidence of anti-LGI1 (leucine-rich, glioma-inactivated 1) encephalitis was 0,83 per million, similar to other neurological diseases such as Creutzfeldt-Jakob Disease and Lambert-Eaton myasthenic syndrome^8^.

In Olmsted County, Minnesota, USA, a recent population-based comparative study showed that prevalence and incidence of autoimmune encephalitis is comparable to infectious encephalitis (13.7/100,000 for autoimmune encephalitis and 11.6/100,000 for all infectious encephalitis)^9^. The same study also demonstrated that detection of autoimmune encephalitis is increasing over time: incidence increased from 0,4/100,000person-years from 1995-2005 to 1.2/100,000 person-years from 2006-2015, attributable to increased detection of autoantibodies-positive cases^9^.

Growing awareness of autoimmune encephalitis has led to adaption of previously proposed criteria for encephalitis (any cause or idiopathic) that focused on infectious causes. Required diagnostic criteria include changes in level of consciousness, fever, CSF (cerebrospinal fluid) pleocytosis, Magnetic Resonance Imaging (MRI) and EEG changes. Nowadays, it is well established that patients with AE without fever can present with memory deficits or behavioral changes without a decreased level of consciousness or fever, and normal CSF (cerebrospinal fluid) examination and brain Magnetic Resonance Imaging (MRI)^2^.

Autoimmune encephalitis can occur in individuals of all ages, some, such as anti-NMDAR (N-methyl-d-aspartate-receptor) encephalitis, predominantly affecting children and young adults. 

## EVOLVING CONCEPTS: NAMING NAMES

Terms such as autoimmune encephalitis, limbic encephalitis, paraneoplastic neurological syndromes are frequently used interchangeably. Although not synonyms, their relationship reflects the evolving knowledge in neuroimmunology. 

The term *paraneoplastic* comes from the Greek, *para=*alongside or near, *neo*=new, and *plasis*=formation. It was not introduced until the mid-1950s and was not widely used in English literature until the 1970s. A broad definition of paraneoplastic syndromes (applicable to neurology and other specialties) refers to disorders caused remotely by cancer, i.e. not by a direct result of cancer invasion of the affected tissue or organ^10^. In the case of paraneoplastic neurologic syndromes, the remote effect is due to an immune-mediated mechanism. 

The beginnings of autoimmune encephalitis occurred in the 1960s and 1970s with the characterization of distinct syndromes associated with cancer. Those syndromes are termed “classical” paraneoplastic syndromes (Table 1) and examples include: encephalomyelitis, limbic encephalitis, rapidly progressive cerebellar syndrome, Opsoclonus-myoclonus, Sensory neuronopathy, Gastrointestinal pseudo-obstruction (enteric neuropathy), Lambert-Eaton myasthenic syndrome. The finding of these specific symptoms or constellations of symptoms strongly suggest the presence of an underlying cancer. 

Among those remarkable syndromes lies limbic encephalitis, described in 1960 by Brierley in the paper “Subacute encephalitis of later adult life. Mainly affecting the limbic areas”, characterized by subacute onset of episodic memory loss, temporal lobe seizures and behavioral abnormalities. In all three studied patients, there were marked inflammatory changes in the medial temporal lobes compared to other sites^11^, supporting the first evidence of an immune-mediated mechanism. Although, inflammation is the main feature of neurological paraneoplastic syndromes, at that time this finding still lacked further supporting evidence. 

It was not until the 1980s that a new surge of interest in paraneoplastic neurological syndromes occurred, with the discovery of antibodies against intracellular neuronal epitopes associated with those diseases. These antibodies are known by the terms onconeural or paraneoplastic antibodies, and examples include anti-Yo, anti-Hu, anti-Ri, and anti-Ma2. Curiously, this nomenclature began to be applied at the Memorial Sloan-Kettering Cancer Center and refers to the initial two letters of the last name of the index patient, while the nomenclature applied at Mayo Clinic (e.g.anti-PCA-1, anti-ANNA-1) refers to the staining pattern by immunohistochemistry(10). Frequently this dual nomenclature causes confusion among clinicians. 

Updated diagnostic criteria for paraneoplastic neurological syndromes have been recently published, and reappraised the three main features of paraneoplastic neurological syndromes considering recent discoveries^12^:

- Paraneoplastic neurologic syndromes can affect any part of the central nervous system, often presenting with stereotyped clinical manifestation. Although there is no pathognomonic neurological presentation associated with paraneoplastic neurological syndrome, some are very indicative of the presence of cancer (Table 1). The updated nomenclature is therefore “high-risk phenotypes” instead of the previously known “classical paraneoplastic neurologic syndrome;
- Paraneoplastic neurologic syndromes occur in association with cancer. Another important concept regarding paraneoplastic neurological syndromes is that association with cancer does not occur by chance, and generic tumor association alone should be used with caution (if not consistent with the phenotype, antigen expression in the tumor must be demonstrated). The causal association between tumor and neurological phenotype is crucial and can be suggested by (a) epidemiological associations (e.g.; rapidly progressive cerebellar syndrome on postmenopausal women is frequently paraneoplastic, associated with specific tumors such as breast and ovarian cancer); (b) antibody associations; beyond supporting diagnosis of paraneoplastic neurological syndromes, antibodies are important to guide investigation of the underlying tumor type;
- Paraneoplastic neurologic syndromes have an immune-mediated pathogenesis that is supported by the frequent finding of specific neuronal antibodies. Instead of the previously known term - onconeural antibodies - the updated recommended nomenclature “high-risk antibodies” (>70% associated with cancer) and presence of these antibodies should be demonstrated using gold standard techniques.

In his Cotzias Lecture, Dr Josep Dalmau, a prominent leading neurologist in the field of paraneoplastic neurological syndromes, reported how he came across a discovery that radically changed concepts about CNS autoimmunity^13^. Such new discoveries were made using the “clinic-to-lab” approach, the same process he had used to describe paraneoplastic syndromes and antibodies, which consists of a selection of patients with similar symptoms, recent diagnosis of cancer leading to subsequent immunological screening and identification of serum or cerebrospinal fluid antibodies against neuronal proteins also present in tumors. Studying four young women with ovarian teratoma (at that time, a tumor not very often associated with paraneoplastic neurological syndromes), prominent neuropsychiatric symptoms, hypoventilation and response to immunotherapy, and negative CSF studies for all intracellular antibodies known at that time, and by optimizing the technique, he was able to demonstrate that samples provoked a unique pattern of neuropil reactivity, indicating that the epitope was in the cell surface. Afterwards the identity of the antigen was established as the NMDAR(N-methyl-d-aspartate-receptor). Following anti-NMDAR (N-methyl-d-aspartate-receptor) syndrome and antibodies, new diseases and antibodies against cell-surface and/or synaptic proteins were and are still being described. 

As a consequence, the term *autoimmune encephalitis* has been little by little adopted to describe those disorders associated the last class of antibodies, despite the fact that, strictly speaking, autoimmune encephalitis can refer to any given target central nervous system cell (neurons, glial cells: astrocytes, oligodendrocytes and microglia) caused by any given immune mechanism (humoral: antibodies and complement, cellular: B and T cells, innate and adaptive).

It is important to make the distinction between “classical” paraneoplastic neurological syndromes related to intracellular antigens and neurological disorders associated with cell surface antigens. While the former tends almost always to be associated with cancer, show little response to treatment, are non-pathogenic but markers of cytotoxic T-cell response, the latter can affect children and young patients, association with cancer is variable (it may occur in the absence of cancer), frequently responds to immunotherapy, and for many antibodies there is strong evidence, including animal models, to support that their pathogenic role. 

However, concepts continue to expand. A recent Spanish prospective multicentre observational study in a pediatric population showed that, among encephalitic syndromes, antibodies against the glial surface protein MOG (Myelin oligodendrocyte glycoprotein) exceeded the frequency of NMDAR (N-methyl-d-aspartate-receptor) antibodies^14^. In other words, even outside the demyelinating spectrum syndromes such as ADEM (acute disseminated encephalomyelitis), MOG antibodies are not only associated with autoimmune encephalitis in children, but also they are the most frequent biomarkers in this scenario. 

Another interdisciplinary field that is gaining attention is the increased incidence of paraneoplastic neurological syndromes in the era of immune checkpoint inhibitors in cancer patients. Although the introduction of this form of immunotherapy enhances immune response against tumors, there is a breakthrough in oncology, and they are associated with several neurological immune-related adverse effects, some of them paraneoplastic neurologic syndrome^15^.

Besides tumors, another remarkable discovery is that anti-NMDAR NMDAR (N-methyl-d-aspartate-receptor) encephalitis might be triggered after herpes simplex encephalitis. A Spanish case series of 99 patients with herpes simplex encephalitis showed that approximately 25% of patients who were followed developed autoimmune encephalitis in an interval ranging from 2-16 weeks after infectious encephalitis^16^. Children aged four or younger were more likely to present with choreoathetosis, decreased level of consciousness, have a shorter interval between onset of herpes simplex encephalitis and autoimmune encephalitis and a worse outcome at one year compared with patients older than four^17^. 

## CLINICAL MANIFESTATIONS

### General considerations

As stated before, autoimmune encephalitis is a group of diseases, some of them more frequently recognized on clinical grounds, such as anti-NMDAR (N-methyl-d-aspartate-receptor) encephalitis and limbic encephalitis (Table 1 and 2). However, a substantial number of individuals affected by autoimmune encephalitis do not present with a well-defined syndrome^2^. Mostly, symptoms progress rapidly, within days or weeks, although there are a few exceptions: some patients with anti-LGI1 (leucine-rich, glioma-inactivated 1) antibodies, anti-CASPR-2 antibodies and anti-DPPX may have a more insidious course^1^. In some instances, patients can present with prodromal symptoms that are characteristics of certain types of disorders - for example - diarrhea and weight loss in anti-DPPX encephalitis or faciobrachial dystonic seizures (FDBS) in LGI-1 encephalitis. However these features are not pathognomonic and might be absent in some patients^1^ ^,^ ^2^. 

**Table 1.** High risk phenotypes.

High-risk phenotype (formerly classical paraneoplastic neurological syndromes)	Clinical features	Antibody associations	Tumor associations	Differential Diagnosis
Encephalomyelitis (EM)	Clinical dysfunction of multiple levels of nervous system, including peripheral involvement, for example EM with peripheral neuropathy, EM with sensory neuronopathy (SNN)	Hu (also called antineuronal nuclear antibody 1, ANNA-1) or CV2/collapsin response-mediator protein 5 (CRMP5) antibodies	SCLC >> NSCLC, other neuroendocrine tumors, and neuroblastoma	Meningeal carcinomatosis (meningeal enhancement, low glucose or presence of tumoral cells in CSF), Neurosarcoidosis (systemic involvement may be shown by FDG-PET, biopsy showing non-caseating granulomas)
Limbic encephalitis	Diagnostic Criteria (Graus 2016) Diagnosis can be made when all four* of the following criteria have been met: 1 Subacute onset (rapid progression of less than 3 months) of working memory deficits, seizures, or psychiatric symptoms suggesting involvement of the limbic system 2 Bilateral brain abnormalities on T2-weighted fluid-attenuated inversion recovery MRI highly restricted to the medial temporal lobes† 3 At least one of the following: • CSF pleocytosis (white blood cell count of more than five cells per mm3) • EEG with epileptic or slow-wave activity involving the temporal lobes 4 Reasonable exclusion of alternative causes *If one of the first three criteria is not met, a diagnosis of definite limbic encephalitis can be made only with the detection of antibodies against cell-surface, synaptic, or onconeural proteins.	High risk antibodies (frequency of cancer>70%)		Infections (Herpes simplex, Human herpesvirus-6, Neurossyphilis, Whipple) Autoimmune systemic diseases (System Lupus Erythematosus, Sj:ogren, Behçet, Relapsing Polychondritis) Gliomas Lymphoma Status epilepticus Chronic temporal lobe epilepsy
		Hu	SCLC >> NSCLC, other neuroendocrine tumors, and neuroblastoma	
		Ma2	Testicular cancer (young men) and NSCLC in older patient (with both Ma1 and Ma2 positivity)	
		High-Risk Antibodies (30-70% of association with cancer)		
		AMPAR	SCLC and malignant thymoma	
		GABABR	SCLC (Paraneoplastic cases are more commonly observed in elderly men and smokers with associated anti-KCTD16 antibodies. Most young patients are not paraneoplastic)	

		Lower-Risk antibodies (<30%)		
		Caspr2	When phenotype os CASOR2 in LE, almost always non paraneoplastic, but if phenotype is Morvan Syndrome, half is associated with tumor	
		LGI1	Thymoma and neuroendocrine	
		GLYR	Malignant thymoma and Hodgkin lymphoma	
		GAD	SCLC, other neuroendocrine tumors, and malignant thymoma	
		AK5		
Rapidly progressive cerebellar syndrome	Previously known as subacute cerebellar degeneration Rapidly progressive, severe, bilateral, cerebellar symptoms. Sometimes extra cerebellar dysfunction maybe present, including brainstem	Anti-Yo (also known as Purkinje cell antibody -1, PCA-1)		Autoimmune cerebellar ataxia antibodies against GAD (glutamic acid decarboxylase), mGLUR1, GLUK2, antibodies gluten ataxia Cerebellar multiple system atrophy Creutzfeldt-Jakob Disease
		Anti-Ri (ANNA-2, ANNA = antineuronal nuclear antibody)	Breast > lung (SCLC and NSCLC) Breast Cancer in Women and lung cancer in men	
		Tr (DNER - elta/notch-like epidermal growth factor related receptor)	Hodgkin lymphoma	
		Ma2 and/or Ma	Testicular cancer (young men) and NSCLC in older patient (with both Ma1 and Ma2 positivity)	
		KLHL11 (Kelch-like protein 11)	Testicular cancer in young men	
Opsoclonus-myoclonus	Involuntary high frequence chaotic multidirectional saccadic movements + nonrhythmic action myoclonus on trunk, limbs and head. Additional features eg. Cerebellar signs and or encephalopathy may be present	-	50% of OMS in children are paraneoplastic and closely associated with neuroblastoma in adults paraneoplastic etiology accounts for 40% of cases. In adults Patients with breast cancer and OMS usually have Ri (ANNA-2 antibodies)	Idiopathic OMS (younger, prodromal symptoms of viral infection/vaccination, less frequently encephalopathy), drugs (lithium, amiytityline, cocaine, phenytoin with diazepam, phenelzine with imipramine, cyclosporin) Neonatal - transient Celiac disease Stem cell transplant HIV Multiple sclerosis Thalamic hemorrhage
Sensory neuropathy	the diagnosis of classical sensory neuronopathy should be considered if all the following criteria are present: - subacute onset with a Rankin score of at least 3 before 12 weeks of evolution, - onset of numbness, and often pain, - marked asymmetry of symptoms at onset, - involvement of the arms, proprioceptive loss in the areas affected, - and electrophysiological studies that show marked, but not restricted, involvement of the sensory fibers with absent sensory nerve action potentials in at least one of the nerves studied.	Hu	SCLC >> NSCLC, other neuroendocrine tumors, and neuroblastoma	Idiopathic (most frequent etiology, painless, onset on lower limbs) Sjögren ´s syndrome Cisplatin/oxaliplatin treatment (usually 1 month after therapy, “coasting phenomenon - progression of sensory loss even after cessation of chemotherapy
		CV2/CRMP5	SCLC and thymoma	
		amphiphysin antibodies	SCLC and breast cancer	
Gastrointestinal pseudo-obstruction	Recurrent episodes of abdominal pain, distension, constipation without evidence of mechanical obstruction Due to myenteric plexus dysfunction and may be accompanied by other signs of autonomic dysfunction, Sensory neuropathy or encephalomyelitis	Hu	SCLC >> NSCLC, other neuroendocrine tumors, and neuroblastoma	Chagas, Diabetes, Parkinson’s Disease, Scleroderma Mechanical obstruction
Lambert-Eaton Myasthenic Syndrome	Progressive proximal lower limb weakness, progressing to upper limbs, distal muscles and finally ocular and bulbar muscles, majority of patients develop autonomic dysfunction (dry mouth, erectile dysfunction, constipation). Absent muscles reflex, which improve after repeat exercise or maximal voluntary contraction (facilitation). EMG shows incremental response after high-frequency nerve stimulation.	antibodies against P/Q type voltage-gated calcium channels (VGCCs) are present in nearly 90% - not necessary for diagnosis.	Present in paraneoplastic and non paraneoplastic LEMS. DELTA-P score for predicting tumor association 1 point for the presence of each of the following items at or within 3 months from onset: age at onset ≥ 50 years, smoking at diagnosis, weight loss ≥ 5%, bulbar involvement, erectile dysfunction, and Karnofsky performance status lower than 70 (Delta P of 4 or more points correspond to >90% of presence of SCLC.	Idiopathic LEMS Myasthenia gravis
		SOX-1	Strongly associates with SCLC or paraneoplastic syndromes associated with SCLC	

**Table 2.** Demographic information, main clinical features of neural surface antibodies.

Antibody	Median Age/Sex ratio (Male to Female)	Main clinical features	Tumor association
NMDAR	21 (2 months- 85 year)/1:4	Clinical features on children’s presentation are usually with neurological symptoms: seizures and dyskinesias; in adults: behavioral changes.	Varies with age and sex Teratoma in almost 50% of young women (aged between 12-45 year) Rare in children and males (6%) titulaer 2013, Graus 2021 Elderly patients (23%), but usually tumors are carcinomas (titulaer late onset)
LGI1	64 years (31-84 years)/2:1	Limbic encephalitis. subtle focal seizures (66%, autonomic or dyscognitive) and faciobrachial dystonic seizures (FBDS, 47%) mostly occurred before onset of cognitive deficits, hyponatremia (60%). MRI normal in 26% od patients, unilateral hippocampal T2/FLAIR hypersignal in 60%, bilateral in 14% CSF cell count and protein unremarkable in 75%	Malignant thymoma and neuroendocrine (<10% of cases)
CASPR2	66 years(25-77)/9:1	Morvan Syndrome and Limbic Encephalitis Seventy-seven percent of the patients had 3 or more of the following symptoms: encephalopathy (cognitive deficits/seizures), cerebellar dysfunction, peripheral nervous system hyperexcitability, dysautonomia, insomnia, neuropathic pain, or weight loss. May have a more protracted clinical course. Median time to nadir of disease was 4 months, and in 30% of patients in 1 year. Increased T2/FLAIR signal in medial temporal lobes in 45% of patients	Tumor association varies with the syndrome. When clinical is Morvan Syndrome, tumor (usually thymoma) if found in 50% of patients When associated with other syndromes, association is low
AMPAR	56 years (23-81)/1:2.3	Limbic encephalitis, Limbic encephalitis with multifocal or diffuse encephalopathy, in rare cases with prominent psychiatric features Increased T2/FLAIR signal in medial temporal loes in 67% of patients	SCLC and malignant thymoma Presence of tumor is higher when other onconeural antibodies occur simultaneously
GABAbR	61 year (16-77)/ 1.5:1	Limbic encephalitis, prominent seizures Increased T2/FLAIR signal in medial temporal lobes in 45% of patients	SCLC Tumor association is higher in elderly men, smokers and co-occurrence of anti-KCTD16 antibodies
GABAaR	40 years (2 mo-88 years); 1:1	Seizures, confusion, behavioral changes. Encephalitis with frequent status epilepticus Cortical and subcortical multifocal abnormalities	Thymoma, paraneoplastic origin is more frequent in adults (60%) than in children (10%)
mGLUR1	55 year (43-64)/1.3	Subacute cerebellar syndrome	30% most hematologic
mGLUR5	29 year (6-75)/ 1.5:1	Encephalitis, main clinical features are psychiatric (Ophelia Syndrome), cognitive , movement disorders, sleep dysfunction, and seizures MRI abnormalities usually involving extra limbic regions	Hodgkin lymphoma in approximately 50% of patients
DPPX	52 year (13-76)/ 2.3:1	Prodrome with diarrhea and weight loss. Encephalitis with hyperekplexia, myoclonus and tremors	B cell neoplasms(<10%)
Neurexin 3-alfa	44 year (23-57)/2:4	Encephalitis, patients may have history or laboratory findings suggestive of systemic autoimmunity, such as increased antinuclear antibodies (ANA), Raynaud < arthralgias	No associated cancer
GluK2	28 years (14-75 )/1.6:1	Encephalitis with prominent clinicoradiological cerebellar involvement, cases of patients with obstructive hydrocephalus)	Few published cases, 2 patients with tumor (Hodgkin’s lymphoma, ovarian teratoma) In this paper there 5 additional patients that had other antibodies concurrent with Gluk2 antibodies, in those 4 had tumors (3 thymoma, 1 small cell lung cancer)
GlyR	50 year(1-75) /1:1	PERM (progressive encephalopathy with rigidity and myoclonus), Limbic encephalitis	(<10%) Malignant thymoma and Hodgkin lymphoma
MOG	37 year (1-74) 1:1	Msost important biomarker of autoimmune encephalitis in children, other than ADEM spectrum, Imaging may resemble that of GABAaR e anti-dopamine 2 receptor ). Phenotype associated with bilateral cortical involvement and leukodystrophy-like has a poor prognosis In adults, beyond the well known presentation (ADEM, optic neuritis, transverse myelitis, demyelinating brain or brainstem syndromes) patients may have overlap with anti-NMDAR encephalitis or present with a benign, unilateral, cerebral cortical encephalitis with epilepsy	Low risk, only 5 cases reported, mostly teratomas
IgLON5	62 year (42-91) 1,25:1	distinctive sleep disorder in association with one or more of the following symptoms: bulbar dysfunction, gait difficulties, oculomotor abnormalities, chorea, or cognitive deterioration.	

## ANCILLARY TESTS

### Brain magnetic resonance imaging (MRI)

**Figure 1.**
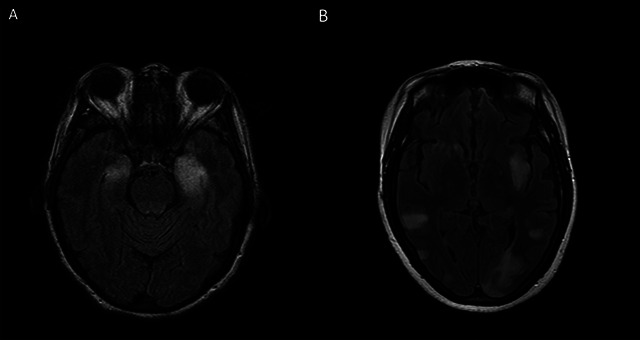
Axial fluid-attenuated inversion recovery (FLAIR) (A,B). A. Increased signal on both medial temporal lobes in a patient with Limbic encephalitis associated with-anti AMPAR antibodies; B. multiple cortical and subcortical FLAIR signal changes involving both hemispheres, without restriction on diffusion sequences in a patient with GABAaR encephalitis Gamma-Amino Butyric Acid type A receptor alpha-amino-3-hydroxy-5-methyl-4-isoxazolepropionic acid receptor.

Regarding complementary tests, magnetic resonance imaging (MRI) is usually normal or shows nonspecific inflammatory changes. However, in two instances MRI findings are specific markers of disease; including GABAaR (Gamma-Amino Butyric Acid type A receptor) encephalitis and limbic encephalitis (Figure 1).

Neuroimaging of GABAaR (Gamma-Amino Butyric Acid type A receptor) encephalitis Fluid-attenuated inversion recovery (FLAIR) sequences show multifocal cortical and subcortical signal abnormalities, without restricted diffusion on Diffusion weighted imaging (DWI) or gadolinium enhancement, mainly distributed in the frontal and temporal lobes, and less frequently in the cerebellum and basal ganglia^18^. As GABAaR (Gamma-Amino Butyric Acid type A receptor) encephalitis can affect children, it is important to be aware of this image pattern, because it may resemble ADEM (acute disseminated encephalomyelitis) ADEM or non-ADEM (acute disseminated encephalomyelitis) encephalitis associated with MOG antibodies^1^ ^,^ ^14^. In limbic encephalitis Magnetic Resonance Imaging (MRI) shows increased signal in T2 and Fluid-attenuated inversion recovery (FLAIR) sequences in the medial aspects of the temporal lobes, frequently unilateral, but images can even be normal.

In addition to the few conditions where imaging findings are very specific and relatively frequent in certain clinical scenarios, Magnetic Resonance Imaging (MRI) is also important to help exclude alternative diagnoses such as stroke, Creutzfeldt-Jakob disease (CJD) and infectious causes. Lesions affecting mesial temporal lobes and beyond (non-mesial temporal lobes, orbitofrontal cortex) associated with parenchymal hemorrhage on susceptibility-weighted imaging (SWI) or with T2*-weighted gradient echo (GRE) should promptly raise suspicions of herpes simplex virus (HSV) encephalitis^19^.

### Cerebrospinal fluid (*CSF*) examination

Examination of Cerebrospinal fluid (CSF) is abnormal in most patients, mostly showing mild lymphocytic pleocytosis (<100 cells per mm^3^), presence of *oligoclonal* bands (OCB) or increased IgG index and/or IgG synthesis rate. Cerebrospinal fluid (CSF), however, can also be normal and absence of pleocytosis does not rule out diagnosis. Anti-LGI1 (leucine-rich, glioma-inactivated 1) encephalitis, for example, frequently presents with normal Cerebrospinal fluid (CSF) findings: cell and protein counts may be unremarkable in up 75% of patients^8^. 

### Electroencephalogram (EEG)

Despite widespread use of electroencephalogram (EEG) to assess patients with suspected autoimmune encephalitis, EEG usually shows non-specific abnormalities. In anti-NMDAR (N-methyl-d-aspartate-receptor) encephalitis, electroencephalogram (EEG) is abnormal in more than 90% of cases, although findings are usually non-specific and include: diffuse slowing in (91% of patients), focal slowing (34% of patients), diffuse excessive beta-activity (52-71% of patients, probably medication related), and Generalized Rhythmic Delta Activity (GRDA) (51% of patients)^20^ ^-^ ^22^. A more specific non-epileptic pattern is extreme delta-brush (EDB). characterized by rhythmic delta activity at 1-3 Hz with superimposed bursts of rhythmic 20-30 Hz beta frequency activity “riding” on each delta wave, and resembling a preterm neonatal EEG pattern known as delta brush^20^. Extreme delta brush (EDB) can be seen in 13%-58% of cases, according to the study, and is not a sensitive finding. 

Data from a French Study with anti-NMDAR (N-methyl-d-aspartate-receptor) encephalitis showed that non-epileptic EEG patterns follow a chronological organization in the disease’s course: the first finding in order of appearance is excessive beta activity (EBA, median time of 10 days), followed by Extreme delta brush (EDB, median time of 16.5 days) and, lastly, Generalized Rhythmic Delta Activity (GRDA, median time of 21.5 days)^22^. The same study also underscores the importance of distinguishing seizures from movement disorders in anti-NMDAR (N-methyl-d-aspartate-receptor) encephalitis. Presence of Generalized Rhythmic Delta Activity (GRDA), a non-epileptic pattern, strongly associated with abnormal movements, may lead to misinterpretation of this finding as seizure related or status epilepticus, which may result in aggressive and unnecessary anti seizure treatment^22^. In other autoimmune encephalitis, such as anti-GABAaR (Gamma-Amino Butyric Acid type A receptor), lateralized periodic discharges (LPDs, previously called periodic lateralized epileptiform discharges - PLEDS) also found in Herpes Simplex Virus (HSV) encephalitis or other acute destructive lesions can also occur^23^. 

Although electroencephalogram (EEG) sensitivity is high, normal EEG does not exclude autoimmune encephalitis. For example; in anti-NMDAR (N-methyl-d-aspartate-receptor) encephalitis electroencephalogram (EEG) is normal in 4% of patients, and in LGI1 (leucine-rich, glioma-inactivated 1), EEG usually shows no eletroencephaloraphic correlate with faciobrachial dystonic seizures (FDBS)^8^. 

Some findings in electroencephalogram EEG may have prognostic value. Extreme Delta Brush (EDB) in anti-NMDAR (N-methyl-d-aspartate-receptor) encephalitis is associated with more prolonged illness and increased number of days of electroencephalogram (EEG) monitoring^20^. Presence of normal posterior rhythm in the initial electroencephalogram (EEG) recording is associated with a better modified Rankin Scale on final outcome^21^.

## BRAIN ^18^FLUORODEOXYGLUCOSE POSITRON EMISSION TOMOGRAPHY (^18^FDG-PET)

Although there is a potential use of Brain ^18^fluorodeoxyglucose positron emission tomography (^18^FDG-PET) in the diagnosis and treatment of autoimmune encephalitis, the lack of specificity and limited availability limits FDG-PET use in the diagnosis of autoimmune encephalitis^19^. 

In anti-NMDAR (N-methyl-d-aspartate-receptor) encephalitis, a pattern of decreased occipital lobe metabolism by ^18^fluorodeoxyglucose positron emission tomography (^18^FDG-PET) can occur, correlating with disease severity^24^ ^,^ ^25^. Bilateral temporal hypermetabolism also favors diagnosis of limbic encephalitis^19^.

However, indiscriminate use of ^18^fluorodeoxyglucose positron emission tomography (^18^FDG-PET) frequently leads to confusion. In clinical practice it is not uncommon that a ^18^fluorodeoxyglucose positron emission tomography (^18^FDG-PET ) report turns out as “suggestive of autoimmune encephalitis”. Hitherto, there are no data validating ^18^fluorodeoxyglucose positron emission tomography (^18^FDG-PET) positive and negative predictive values to diagnose autoimmune encephalitis, nor to differentiate autoimmune encephalitis from neurodegenerative and infectious etiologies. Awareness of some caveats are crucial in interpreting ^18^fluorodeoxyglucose positron emission tomography (^18^FDG-PET) results. All encephalitis, infectious and non-infectious, are frequently associated with seizures and inflammation. In addition, effects of antibodies and medications (e.g., anesthetics, anti seizures, immunosuppressants) might also alter metabolic findings on positron emission tomography (^18^FDG-PET) imaging, potentially limiting this method’s specificity in establishing the etiology of the disorder^19^ ^,^ ^26^. One could draw a parallel with the role of ^18^fluorodeoxyglucose positron emission tomography (^18^FDG-PET) in gliomas. Regardless of the extensive literature on ^18^fluorodeoxyglucose positron emission tomography (^18^FDG-PET) in gliomas, it has not been incorporated into most widely accepted criteria to assess response of therapy^26^.

### Clinical Scenarios

### Clinical scenarios and controversies

Over the past years, ongoing discovery of novel disorders associated with antibodies against cell surface or synapsis has become of high interest for practicing neurologists because of the opportunity to diagnose and treat previously unknown or mischaracterized conditions. Given the potential treatability of many of these disorders, a high index of suspicion is compelling for low threshold for antibody testing, or labeling patients with an “autoimmune” neurological condition in the absence of strong data that supports the hypothesis. 

Neuroimmunology is not immune to the reproducibility crisis, a current hot topic in science. A *Nature* survey showed that over 70% of researchers were unable to reproduce the findings of other scientists and approximately 60% could not reproduce their own findings^27^. Among shortcomings that impact reproducibility in the field of autoimmune encephalitis, we can find publications regarding neuronal autoantibodies that show important methodological flaws. 

Unfortunately, because of the amount of misinformation, we are letting complementary tests override clinical assessment or labeling patients with autoimmune disease in circumstances where specific biomarkers, distinctive syndromes or neuropathological findings are lacking. In the following topics we will discuss some caveats and pitfalls in autoimmune encephalitis. 

### Autoimmune psychosis and first episode psychosis (FEP)

Diagnosis of anti-NMDAR (N-methyl-d-aspartate-receptor) encephalitis in mental health research is challenging for several reasons. In the first place, almost 80% of patients with anti-NMDAR (N-methyl-d-aspartate-receptor) initially present with prominent psychiatric symptoms, including psychosis mimicking a primary psychiatric illness. Another reason is that patients’ antibodies are pathogenic and cause hypofunction of NMDAR (N-methyl-d-aspartate-receptor), a key role in pathophysiology of schizophrenia. Consequently is its imperative to ask: When should we suspect autoimmune encephalitis in patients with first episode psychosis (FEP)? Should we test every patient with first episode of psychosis (FEP) for neuronal antibodies? Is schizophrenia a primary psychiatric disorder or could it have an underlying autoimmune basis?

To start answering this question it is crucial to keep in mind that anti- NMDAR (N-methyl-d-aspartate-receptor) rarely presents with isolated isolated psychiatric manifestations (approximately 4%, most of patients during diseases relapses) . the disease usually presents with a marked constellation of syndrome that clinically suggests the diagnosis (Table 3). Moreover in anti- NMDAR (N-methyl-d-aspartate-receptor), antibodies specific to the GluN1 subunit present in CSF cerebrospinal fluid) are of immunoglobulin G (IgG) class, detectable by techniques that preserve the native conformation of epitopes., namely: the cell-based assays (used by most clinical laboratories), immunohistochemistry of brain sections (commercially available; sometimes used as a confirmatory test), and immunocytochemistry of cultures of dissociated rodent live hippocampal neurons (only used in research laboratories)^2^. 

**Table 3.** Diagnostic criteria of anti-NMDAR encephalitis (Adapted from Dalmau et al., 2019).

Probable
Rapid onset (<3 months) of at least four of the six major groups of symptoms: Group 1: Psychiatric symptoms: abnormal(psychiatric) behavior or cognitive dysfunction Group 2: Language: Speech dysfunction ( pressured speech, verbal reduction, or mutism) Group 3: Seizures Group 4: Movement disorders: dyskinesias, rigidity, or abnormal postures Group 5: Decreased level of consciousness Group 6: Autonomic dysfunction or central hypoventilation
And at least one of the laboratory studies: Abnormal EEG (focal or diffuse slow or disorganized activity, epileptic activity, or extreme delta brush) CSF with pleocytosis or oligoclonal bands
Or 3 of the above groups of symptoms and identification of a systemic teratoma
Exclusion of recent history of herpes simplex virus encephalitis or Japanese B encephalitis, which might result in relpasong immune-mediated neurological symptoms
Definite
One or more of the six major groups of symptoms and IgG GluN1 antibodies (antibody testing should include CSF); if only serum is available, confirmatory tests should be included (eg, live neurons or tissue immunohistochemistry, in addition to a cell-based assay)
Exclusion of recent history of herpes simplex virus encephalitis or Japanese B encephalitis, which might result in relapsing immune-mediated neurological symptoms

A considerable number of publications postulating that the same antibodies that are associated with anti- NMDAR (N-methyl-d-aspartate-receptor also occur in primary psychiatric diseases have been published. The term *autoimmune psychosis*, emulating the term *autoimmune encephalitis*, was coined to refer to schizophrenic patients or patients with the first episode of psychosis (FEP) suspected of being autoimmune in origin, leading to proposed immunotherapy treatment. Unfortunately, many of these publications have important methodological flaws^28^ ^,^ ^29^. There is a very low prevalence (approximately 1%) of NMDAR (N-methyl-d-aspartate-receptor) -antibodies and other neuronal antibodies in the serum of patients with schizophrenia and other psychiatric diseases. If CSF (cerebrospinal fluid) is examined, it is remarkably negative^29^. As mentioned above, most studies of neuronal antibodies’ prevalence in these patients were performed in serum using techniques with suboptimal specificity, that were not validated. Moreover, in some papers, the Ig class against NMDAR (N-methyl-d-aspartate-receptor) was IgA and IgM which are not clinically relevant, errouneously indicating a prevalence of clinically relevant antibodies. So far, no study has been able to demonstrate a diagnostic relevance or therapeutic meaning in a well-defined group of autoimmune psychosis or NMDAR (N-methyl-d-aspartate-receptor) detection in the serum of patients with schizophrenia or first episode of psychosis (FEP).

Because of fear of misdiagnosing (and consequently, delaying treatment) patients with emerging psychiatric symptoms, a practicing neurologist’s assessment is often requested in this scenario. It is important to keep in mind that even though the majority (>85%) of patients with anti-NMDARE (N-methyl-d-aspartate-receptor encephalitis) present with behavior changes, agitation, hallucinations, delusions or catatonia, most of which (>90%) will progress to neurological symptoms. Furthermore, 95% of patients have abnormal EEG (electroencephalogram) findings, 55% have abnormal MRI (magnetic resonance imaging) and 80% show CSF (cerebrospinal fluid) changes, making the diagnostic process easier^30^. Arguing that it would facilitate diagnosis of psychosis of autoimmune origin in patients with isolated or predominant psychiatric symptoms, some authors have recycled the typical neurological and paraclinical findings present in autoimmune encephalitis to propose diagnostic criteria for the so-called *autoimmune psychosis* ^31^. These criteria profoundly depend on the presence of neurological symptoms, and they work better when they are less needed: in clinical settings where patients present with conspicuous features of autoimmune encephalitis. On the contrary; those criteria are fallible in the rare instances of pure psychiatric manifestations, when they would be truly needed^28^. To illustrate, a recent study with 103 patients with first episode psychosis (FEP) found that 34 (32%) and 39 (37%) patients fulfilled two sets of warning signs of *autoimmune psychosis* and 21 (20%) fulfilled the criteria for possible or probable *autoimmune psychosis*, despite none of these patients had psychosis of autoimmune origin. Notably, the same criteria missed diagnosis of two out of three patients with anti-NMDAR encephalitis who were the only ones with psychosis of autoimmune origin(28). Such findings call into question the ability to identify patients with first episode due to autoimmune encephalitis by the *autoimmune psychosis* ^31^ criteria alone^32^. 

The same study tried to shed light on the selection of at-risk patients for lumbar puncture, a procedure that may be difficult in psychiatric patients with agitation. By this approach, lumbar puncture would be indicated in only 27% of patients with first episode psychosis^28^. So, how should patients with recent (<6 months) first episode psychosis (FEP) without neurological features or findings be assessed? The authors elaborated an algorithm^28^:

- All patients with first episode psychosis (FEP) with accompanying neurological symptoms and abnormal paraclinical tests such as EEG (electroencephalogram) and Brain MRI (magnetic resonance imaging) or unclear etiology should undergo lumbar puncture;
- Patients without neurological changes, serum antibody testing, EEG electroencephalogram) and Brain MRI (magnetic resonance imaging) should be ordered. If any test is abnormal, CSF (cerebrospinal fluid) is warranted, including cell-count, oligoclonal bands and NMDAR (N-methyl-d-aspartate-receptor) -antibodies testing;
- If serum is negative for NMDAR (N-methyl-d-aspartate-receptor) - antibodies, presence of any features associated with NMDARE (N-methyl-d-aspartate-receptor encephalitis), namely: abnormal EEG (electroencephalogram) and/or Brain MRI (magnetic resonance imaging) findings, resistance or adverse reactions to neuroleptics, subsequent development of neurological symptoms and presence comorbid conditions such as prodromal viral-like illness, tumor), CSF (cerebrospinal fluid) testing is also warranted. This is necessary because approximately 15% of patients have absent anti-NMDAR (N-methyl-d-aspartate-receptor) in serum. However, the absence of antibodies in CSF (cerebrospinal fluid) excludes antibody mediated first episode psychosis (FEP);
- In first episode psychosis (FEP) with isolated psychiatric features detection of other antibodies in CSF (cerebrospinal fluid) other than anti-NMDAR (N-methyl-d-aspartate-receptor) is extremely rare. Testing for other antibodies should be considered if they are found in serum or if CSF (cerebrospinal fluid) anti-NMDAR (N-methyl-d-aspartate-receptor) are negative.

However, this study has several limitations, mainly because it is modest in size, so larger studies and meta-analyses are required to establish the prevalence of anti-NMDARE (N-methyl-d-aspartate-receptor encephalitis) and optimize the selection of patients for CSF (cerebrospinal fluid) sampling^32^. In our experience of managing such cases in a clinical scenario where antibody testing is limited or the results may take several weeks to be available, close and thorough observation together with psychiatrists periodically assessing for the emergence of neurological features is very useful in not misdiagnosing AE.

### Autoimmune encephalitis resembling dementia syndromes

Autoimmune encephalitis can mimic neurodegenerative dementia syndromes, as patients may not always present with encephalitis biomarkers (e.g.: brain imaging or cerebrospinal fluids suggestive of inflammation).

The majority of patients with cognitive decline associated with autoimmune encephalitis fulfill diagnostic criteria for autoimmune encephalitis that requires subacute deterioration of cognition, altered mental status or psychiatric features. Other distinctive neurological manifestations such as seizures, new focal neurological signs along with biomarkers of central nervous system inflammation, such as CSF (cerebrospinal fluid) pleocytosis or brain MRI (magnetic brain imaging) changes may also aid in raising suspicion of an autoimmune cause. 

However, there are some instances where encephalitis signs may be more inconspicuous, resembling neurodegenerative dementia syndromes, leading to misdiagnosis or delay in treatment, that may result in worse outcomes.

In order to address the question of autoimmune encephalitis resembling dementia syndromes, a nationwide observational cohort study in middle-aged and older patients (>45 year-old) with anti-LGI1 (leucine-rich, glioma-inactivated 1), anti-NMDAR, anti-GABAbR (Gamma-Amino Butyric Acid type A receptor), and anti-CASPR2 encephalitis was conducted in the Netherlands, with interesting findings and lessons to be drawn^33^.

First, autoimmune encephalitis frequently resembles dementia, especially in the presentation of rapidly progressive dementia. Cognitive decline was the presenting symptom in most patients > 45 years old with antibody mediated encephalitis (75%), in half of these cases, a neurodegenerative dementia syndrome was suspected by the treating physician. Remarkably, cognitive domains are affected in different manners according to the antibody associated syndrome. Visuospatial and executive functions were more prominently affected in LGI-1(leucine-rich, glioma-inactivated 1) and GABA_b_R (gamma-aminobutyric acid B receptor) encephalitis, while patients with anti-NMDARE (N-methyl-d-aspartate-receptor encephalitis) show more frequently language function impairment and behavioral changes. 

Second, seizures are an important red flag to differentiate between a possible autoimmune encephalitis, when patients present with dementia symptoms. Seizures can be subtle and can appear late in the course of the disease. Early and overt seizures in patients with dementia promptly raise the suspicion of a non-degenerative cause, although 10-22% of patients with Alzheimer’s Disease may develop seizures during the course of the disease. Even with the exclusion of patients with prominent seizures within the first four weeks of symptoms presentation (that is, a scenario where autoimmune encephalitis is more easily considered), the study showed that two-thirds of patients developed seizures later on and they were often overlooked in a quarter of patients because seizures were subtle. Seizures were more frequently, and almost exclusively, seen in anti-LGI1 (leucine-rich, glioma-inactivated 1) encephalitis, and were consisted of faciobrachial dystonic seizures (FBDS) and nonmotor focal subtle seizures^33^. Faciobrachial dystonic seizures (FBDS) is a very specific finding for anti-LGI1 encephalitis, defined by some authors as pathognomonic^34^ ^,^. and characterized as frequent (it is not rare for patients to present more than 100 episodes a day), brief events with posturing of the ipsilateral face and arm, that also involve the leg^35^. Seizures usually do not respond to antiseizure medication, frequently ictal EEG (electroencephalogram) shows no ictal correlates, and seizures are responsive to immunotherapy. 

Recently, ancillary testing has been found to be deceptively normal in many cases. Normal routine CSF (cerebrospinal fluid) and brain MRI (magnetic resonance imaging) were found in more than 50% of patients, and neither CSF pleocytosis nor MRI (magnetic resonance imaging) inflammatory changes were found in 25% of patients. Electroencephalograms (EEG) was also frequently normal or only showed some encephalopathy, similar to patients with neurodegenerative dementia. Interestingly, the study demonstrated that dementia biomarkers can be “falsely” positive in AE patients. In almost half of patients with autoimmune encephalitis tested with biomarkers (among whom Aβ42 was also tested), the combination fitted a profile of neurodegenerative dementia. A few cases of autoimmune encephalitis had a positive 14-3-3. Samples analyzed by RT-QuIC - a more specific marker for prion disease - were all negative. 

## TREATMENT

There are three mainstays in treating patients with autoimmune encephalitis: 01) immunotherapy; 02) removal of the immunological trigger, i.e.: tumor when applicable; and 03) symptomatic treatment and rehabilitation. 

Regarding immunotherapy, recommendations are largely drawn from retrospective series and expert opinions. This involves escalation of immunotherapy, beginning with first line therapies (corticosteroids, intravenous gamma globulins, or plasma exchange), second-line therapies (rituximab and or cyclophosphamide). Intravenous gamma globulin and plasma exchange act by removing circulating blood antibodies, while rituximab would eliminate B cells, reducing their role as antigen presenting cells, reducing antibody production and preventing subsequent development of plasma cells. In turn, corticosteroids and cyclophosphamide have a role in decreasing inflammatory infiltrates and production of pro-inflammatory cytokines.

One important concept is that the immune targets of autoimmune encephalitis are located beyond the blood brain-barrier, and that many patients have compartmentalization of the autoimmune process with intrathecal synthesis of antibodies by plasma cell within brain and meninges^36^, which in part explains limited effectiveness of plasma exchange and intravenous immunoglobulin in comparison to systemic antibody mediated diseases as myasthenia or immune thrombocytopenic purpura^1^.

Other factors dictating immunotherapy strategy, such as response to corticosteroids, speed of recovery, degree of residual deficits and risk of relapse varies according to the antibody associated with disease, and further data are needed to tailor immunotherapy. For example: patients with anti-LGI1 (leucine-rich, glioma-inactivated 1) encephalitis seem to respond better to corticosteroids, have a faster recovery, although they tend to remain with significant residual cognitive deficits^8^ ^,^ ^37^ in comparison to patients with anti-NMDAR (N-methyl-d-aspartate-receptor) encephalitis, who have a poorer response to first line therapy, longer ICU and hospital stay, and time to recovery, but with majority of patients achieving good outcomes^30^.

Anti- NMDAR (N-methyl-d-aspartate-receptor) encephalitis is more frequent and more studied than antibody-associated encephalitis. Approach to anti-NMDAR encephalitis is based on a study of 472 patients that showed that no improvement at four weeks of first-line therapy; which is frequent and occurs in about 50% of patients. Among non responders, patients who received second-line therapies had a better outcome after 24 months, compared with patients that did not receive second-line treatment^30^. Because anti-NMDAR encephalitis occurs more commonly in women with child-bearing potential, we prefer rituximab over cyclophosphamide, to avoid the risk of cyclophosphamide induced gonadal toxicity.

In general, patients are observed at two week intervals, and if there is minimal or no response, treatment is escalated to second-line therapy^35^. Because patients who receive rituximab have a lower chance of relapse, some authors consider this drug in initial therapy, although there are no studies to support this use. 

Approximately in 10% of patients with anti-NMDAR (N-methyl-d-aspartate-receptor) encephalitis, disease will be refractory to first and second-line therapy^30^. For these patients some authors have suggested treatment with bortezomib (a proteasome with anti-plasma cell activity)^38^ ^-^ ^40^, tocilizumab (an interleukin-6 receptor agonist)^41^ and intrathecal or oral methotrexate^42^ ^,^ ^43^. However, studies supporting such an approach have important limitations: small number of patients, use of other immunotherapies and short period considered to define failure to second-line therapy, and these studies should be interpreted with caution. 

An ongoing trial, ExTINGUISH (The ExTINGUISH Trial of Inebilizumab in NMDAR Encephalitis) will randomize 116 participants with moderate-to-severe NMDAR encephalitis to receive either inebilizumab or placebo in addition to first-line therapies^44^. Inebilizumabe is a monoclonal antibody against the B-cell surface antigen CD19, created to treat neuromyelitis optica spectrum disorder. Compared to other therapies targeting B-cell depletion (e.g.: rituximab), inebilizumab depletes CD 20+ and CD20- plasma cells and plasma blasts, that may play a role in refractory anti-NMDAR encephalitis. 

In conclusion, the discovery of this new class of autoimmune encephalitis has dramatically changed the diagnostic approach and treatment of many neurological syndromes, some of which remain completely unknown. 

Unfortunately, despite clinical advances, many studies have important limitations, and there is an urgent need for rigorous clinical and immunological criteria to diagnose autoimmune encephalitis, to minimize misdiagnosis. In addition a better comprehension of each antibody associated with encephalitis is needed to tailor immunotherapy. Development of new therapeutic strategies are needed to improve outcomes and to speed recovery rate.
